# Targeting the *Mycobacterium tuberculosis* transpeptidase Ldt_Mt2_ with cysteine-reactive inhibitors including ebselen†
†Electronic supplementary information (ESI) available: Experimental details, IC_50_ curves, mass spectra, electron density maps. See DOI: 10.1039/c9cc04145a


**DOI:** 10.1039/c9cc04145a

**Published:** 2019-08-05

**Authors:** Mariska de Munnik, Christopher T. Lohans, Pauline A. Lang, Gareth W. Langley, Tika R. Malla, Anthony Tumber, Christopher J. Schofield, Jürgen Brem

**Affiliations:** a Chemistry Research Laboratory , Department of Chemistry , University of Oxford , Oxford , OX1 3TA , UK . Email: christopher.schofield@chem.ox.ac.uk ; Email: jurgen.brem@chem.ox.ac.uk; b Department of Biomedical and Molecular Sciences , Queen's University , Kingston , ON K7L 3N6 , Canada

## Abstract

The l,d-transpeptidases (Ldts) are promising antibiotic targets for treating tuberculosis. We report screening of cysteine-reactive inhibitors against Ldt_Mt2_ from *Mycobacterium tuberculosis*. Structural studies on Ldt_Mt2_ with potent inhibitor ebselen reveal opening of the benzisoselenazolone ring by a nucleophilic cysteine, forming a complex involving extensive hydrophobic interactions with a substrate-binding loop.

## Abstract 2

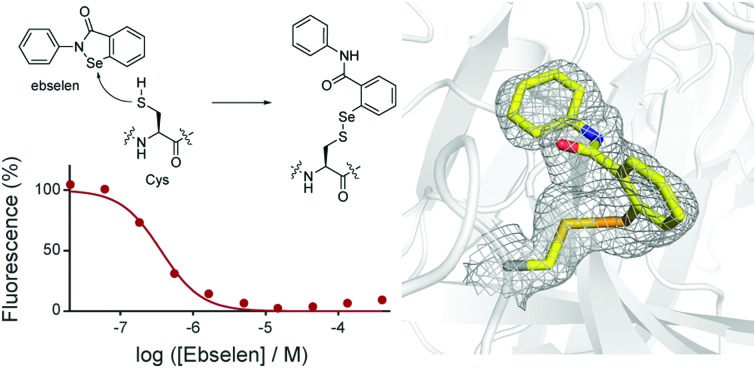
Inhibitors targeting the conserved nucleophilic cysteine of the mycobacterial l,d-transpeptidases are a potential strategy for the treatment of tuberculosis.

## 

Tuberculosis (TB) is the leading cause of death associated with a single infectious agent worldwide.^1^ Whilst current TB therapies can be effective, the treatment duration, the need for co-administration of multiple drugs, side effects, and limited drug availability in developing countries hinders successful treatment in many highly-affected regions.^1^ In recent years multi-drug resistant (MDR) and extensively-drug-resistant (XDR) strains of the causative agent, *Mycobacterium tuberculosis*, have emerged.^2^ There is thus a pressing need for improved and inexpensive TB therapies which target resistant strains and which require a shortened treatment duration.

The cell wall peptidoglycan of most Gram-negative bacteria consists primarily of 4 → 3 peptide cross-links between *meso*-diaminopimelate (*meso*-Dap) and d-alanine residues (*i.e.*, *meso*-Dap-d-Ala cross-links). The formation of these 4 → 3 cross-links is catalysed by the d,d-transpeptidases (or penicillin-binding proteins; PBPs), which employ a nucleophilic serine residue. The PBPs are the primary targets of the β-lactam antibacterials, which are of immense clinical importance, but which, despite promise,^3^–^5^ have not been developed for clinical use against TB. In *M. tuberculosis*, the cell wall contains high levels of 3 → 3 (*meso*-Dap–*meso*-Dap) cross-links (approx. 80% at stationary phase), which are formed by the l,d-transpeptidases (Ldts) (Fig. S1, ESI†).^6^ Evidence has emerged that Ldt_Mt2_, in particular, plays an important role in the virulence of *M. tuberculosis*, as disruption of the *ldt*_*Mt2*_ gene results in altered morphology and inhibition of colony growth.^7^ Mechanistically, the Ldts differ from the PBPs through their use of a nucleophilic cysteine rather than serine during catalysis.^8^

Several β-lactam antibiotics, in particular carbapenems, have been shown to inhibit the activity of Ldt_Mt2_ and to have anti-TB activity.^3^,^5^,^10^–^12^ Such Ldt_Mt2_ inhibition involves covalent modification of the nucleophilic cysteine (Cys354), which reacts with β-lactam antibiotics to give (a) stable acyl–enzyme complex(es) (Fig. 1A).^5^,^10^,^11^ Despite the promise associated with the treatment of TB using β-lactams, their application is hindered by cost, stability, and delivery issues (due in part to the need to target *M. tuberculosis* present in macrophages). There is therefore interest in developing alternative ways of inhibiting the Ldts and, more generally, mycobacterial transpeptidases (including PBPs). As targeting nucleophilic cysteine residues is a validated method for inhibitor development for human intracellular targets,^13^–^15^ we were interested in exploiting such an inhibition strategy for the treatment of TB. Here we report the application of a fluorescence-based assay for Ldt_Mt2_ for the identification of cysteine-reactive reagents, including the drug candidate ebselen,^16^ as promising inhibitors of the Ldts.

**Fig. 1:**
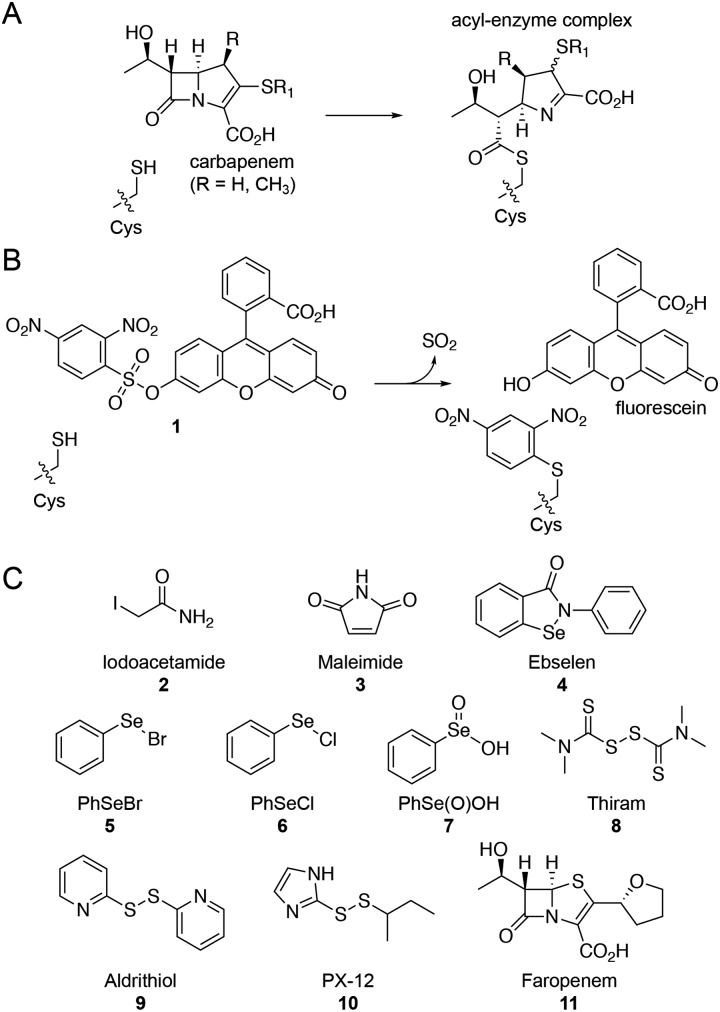
Ldts are targets for the treatment of *M. tuberculosis.* (A) Reaction of the Ldt_Mt2_ nucleophilic cysteine with a carbapenem β-lactam antibiotic to form a stable acyl–enzyme complex. (B) Proposed reaction of the Ldt_Mt2_ nucleophilic cysteine with fluorogenic probe **1**,^9^ releasing SO_2_, fluorescein, and arylating the cysteine residue. (C) Structures of the cysteine-reactive reagents tested for inhibition of Ldt_Mt2_. Faropenem (**11**) was included as a positive control.

Due to the limitations associated with spectrophotometric assays for the Ldts (*e.g.*, poor sensitivity and high protein requirements),^5^,^10^ we devised an alternative fluorescence-based assay for screening Ldt_Mt2_ inhibitors (manuscript in preparation).^17^ Although a number of fluorogenic probes selective for small molecule thiols (*e.g.*, cysteine) are reported,^9^,^18^,^19^ to our knowledge they have not been applied with the Ldts for assay development. We chose to focus on the 2,4-dinitrobenzenesulfonyl fluorescein probe **1** (Fig. 1B), due to the ease of synthesis and the strong fluorescent signal associated with the fluorescein fluorophore.^9^

We applied this assay to study cysteine-reactive reagents as potential Ldt_Mt2_ inhibitors (Fig. 1C). Commonly used methods for the covalent modification of cysteine include thiol-reactive groups as seen in the haloacetyl reagent iodoacetamide (**2**) and the Michael acceptor maleimide (**3**).^20^ Moreover, sulphur- and selenium-based compounds are known for their ability to interact with cysteine residues;^15^,^21^,^22^ therefore, compounds **4–10** (including ebselen) were included in the screen. The penem faropenem (**11**), a known inhibitor of Ldt_Mt2_, was used as a positive control.^5^,^10^

Although compounds **2–11** were all observed to inhibit Ldt_Mt2_ (Table 1 and Fig. S2, ESI†), substantial variations in their activities were observed. IC_50_ values were dependent on incubation time, consistent with a covalent inactivation mechanism. Ebselen (**4**) was the most potent inhibitor identified, with an IC_50_ of 0.36 μM with no pre-incubation, and an IC_50_ of 0.143 μM following 60 minutes of pre-incubation; both IC_50_ values are significantly smaller than those obtained for the positive control faropenem (**11**) under similar conditions (*i.e.*, IC_50_ of 0.686 μM following a 60 minute pre-incubation).

**Table 1:** The inhibitory activity of compounds **2–11** with Ldt_Mt2_

	Compound	IC_50_ (μM, mean ± SD)		
		0 min^*a*^	10 min	60 min
**2**	Iodoacetamide	129 ± 14	32.5 ± 1.4	10.1 ± 0.7
**3**	Maleimide	158 ± 9	48.3 ± 3.6	18.6 ± 0.7
**4**	Ebselen	0.36 ± 0.02	0.159 ± 0.077	0.143 ± 0.014
**5**	PhSeBr	11.8 ± 1.3	5.05 ± 0.35	2.02 ± 0.61
**6**	PhSeCl	62.8 ± 2.2	24.6 ± 0.9	20.0 ± 1.2
**7**	PhSe(O)OH	309 ± 7	136 ± 17	93.2 ± 9.3
**8**	Thiram	7.01 ± 0.29	2.93 ± 0.11	0.780 ± 0.029
**9**	Aldrithiol	22.1 ± 0.7	5.35 ± 0.21	1.61 ± 0.03
**10**	PX-12	27.2 ± 0.5	9.56 ± 0.25	2.48 ± 0.10
**11**	Faropenem	1.69 ± 0.17	1.00 ± 0.04	0.686 ± 0.069

^*a*^The impact of inhibitors on Ldt_Mt2_ was tested without pre-incubation, or with 10 min or 60 min pre-incubation prior to addition of fluorogenic probe **1**.

Ebselen has been shown to be an effective cysteine-targeting reagent in previous studies, *e.g*., inhibiting the enzymes γ-butyrobetaine hydroxylase and JMJD2A through interaction with cysteine residues.^16^,^21^–^23^ In addition, ebselen has been in late stage clinical development as a treatment for stroke,^24^,^25^ and is currently being investigated for the treatment of bipolar disorder and hearing loss.^26^,^27^ The other selenium-containing compounds tested, *i.e.*, **5–7**, were significantly less active than ebselen (Table 1). The sulphur-based compounds **8–10** were also not as potent as ebselen, but following a 1 hour pre-incubation period, all manifested substantial inhibition; in particular, thiram (**8**) was the most potent of these compounds, and showed inhibition comparable with that of faropenem (**11**). The activities of both iodoacetamide (**2**) and maleimide (**3**) were highly dependent on the length of the pre-incubation period, indicating slow reaction with Ldt_Mt2_.

Having shown that the inhibitory activity of compounds **2–11** is time dependent, we investigated their interaction with Ldt_Mt2_ using protein mass spectrometry (MS; Table S1, ESI†). Mass spectra obtained for Ldt_Mt2_ treated with a 10-fold excess of compounds **2–11** showed the formation of adducts consistent with reaction with a single molecule of inhibitor under these conditions. Maleimide (**3**) and ebselen (**4**) appeared to react with Ldt_Mt2_ without fragmentation, while reaction with iodoacetamide (**2**), PhSeBr (**5**) and PhSeCl (**6**) was accompanied with the loss of the corresponding halide. PhSe(O)OH (**7**) reacted to form a similar adduct as **5** and **6**. The molecular weights of the adducts formed with thiram (**8**), aldrithiol (**9**) and PX-12 (**10**) indicated that these inhibitors likely react *via* disulphide exchange.

We then used high-throughput mass spectrometry to examine the rate of adduct formation for Ldt_Mt2_ with two equivalents of inhibitor (Fig. S3 and S4, ESI†). While ebselen (**4**) fully reacted with the enzyme within 1 min of addition, the other selenium-containing compounds investigated (*i.e.*, **5–7**) reacted much more slowly (Fig. S3, ESI†). Complete reaction between Ldt_Mt2_ and aldrithiol (**9**) was observed within 10 minutes. Although thiram and the other sulphur-based compounds (**8–10**) reacted more slowly, complete reaction was observed in all cases within 45 minutes (within detection limits). Similar results were obtained with iodoacetamide (**2**). In agreement with the high degree of time dependence observed for it in the dose–response analysis, maleimide (**3**) reacted relatively slowly, with complete reaction not being observed by 82 minutes. The covalent complexes were observed to be stable for 24 hours, with the exceptions of those derived from thiram and faropenem, for which a small amount of unbound protein was observed by RapidFire MS after this time (Fig. S3, ESI†).

To investigate the structural basis of Ldt_Mt2_ inhibition by ebselen, we carried out crystallographic studies. Ldt_Mt2_ crystallized in the *P*12_1_1 space group with two protein chains in the asymmetric unit (ASU); the structure was solved by molecular replacement using PDB ; 5DU7 5 as a search model (Table S2, ESI†). Consistent with previous reports, the structure of Ldt_Mt2_ consists of two N-terminal immunoglobulin fold-related domains and a C-terminal catalytic domain (Fig. 2A).^11^,^28^ The overall fold of Ldt_Mt2_ in our structure aligns well with reported Ldt_Mt2_ structures,^11^,^28^ with a root-mean-square-deviation of 0.70 Å for backbone Cα atoms compared to PDB entry ; 5D7H.^29^

**Fig. 2:**
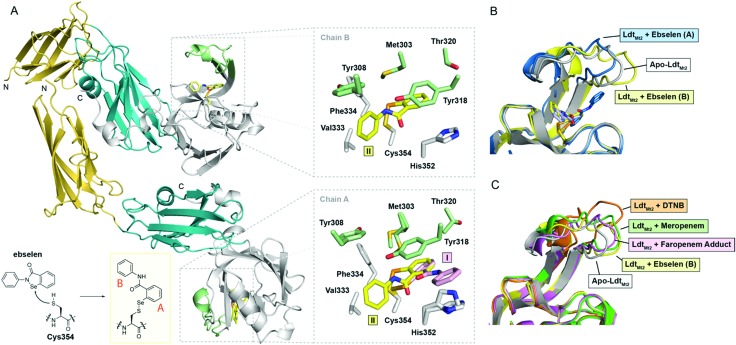
Crystallographic studies of Ldt_Mt2_ with ebselen. (A) View from the crystallographically observed structure of Ldt_Mt2_ in complex with ebselen. The two immunoglobulin-related domains are in yellow and blue, while the catalytic domain is in white; the active-site loop region (lid) of the catalytic domain (residues 300–323) is in green. The inset shows the expected complex formed from ebselen and Cys354. Also shown are views of the active sites of chains A and B, with sticks coloured according to the cartoon representation, highlighting the two ebselen conformations observed (**I**, **II**). (B) Structural alignment of chains A and B of the complex derived from ebselen and Ldt_Mt2_ (blue and yellow cartoons, respectively) with the apo-enzyme (white cartoon), highlighting variations in the active site lid. (C) Overlay of Ldt_Mt2_ complex structures, showing variations in the active site lid. The unmodified enzyme (white cartoon) and ebselen adduct (chain B; yellow cartoon) structures are overlaid with Ldt_Mt2_ complexes derived from 5,5′-dithiobis-(2-nitrobenzoic acid) (DTNB; orange cartoon; PDB ; 5LB1),^10^ meropenem (green cartoon; PDB ; 3VYP),^11^ and faropenem (which fragments to form a 3-hydroxybutyryl group; pink cartoon; PDB ; 5LBG).^10^

Ldt_Mt2_ crystals were soaked with ebselen, and the structure of the complex was solved by molecular replacement (Table S2, Fig. 2A and Fig. S5, ESI†). In both protein chains in the ASU, electron density consistent with the presence of a single ebselen-derived adduct was observed extending from Cys354 (Fig. 2A). While a single ebselen conformation was refined in chain B (conformation **II**), two different conformations were refined in chain A (conformations **I** and **II**). The ratio of conformations **I** and **II** in chain A appeared to depend on the length of time that Ldt_Mt2_ crystals were soaked with ebselen, with the conformation corresponding to that observed in chain B (*i.e.*, conformation **II**) predominating at longer time points (Fig. 2A) (data not shown).

Extensive hydrophobic interactions are apparent between both aromatic rings of ebselen and the Ldt_Mt2_ active site in conformations **I** and **II** (Fig. 2A). In addition to Val333, Phe334 and His352, residues from the mobile active-site loop (a two-stranded β-sheet encompassing residues 300–323) including Met303, Tyr308, Tyr318, and Thr320, contribute to the hydrophobic pocket around Cys354 (Fig. 2A). Apparent pi-stacking between the phenol ring of Tyr318 and the ebselen-derived phenyl ring proximal to Cys354 (ring A in Fig. 2A) is present in conformations **I** and **II**; this interaction has also been observed in the complex derived from Ldt_Mt2_ and 5,5′-dithiobis-(2-nitrobenzoic acid).^10^ The position of the ebselen-derived phenyl ring distal to Cys354 (ring B) depends on the conformation of ebselen (Fig. 2A); in conformation **I**, ring B appears to pi-stack with His352, while in conformation **II**, it interacts primarily with Val333.

The position of the active-site lid (residues 300–323; Fig. 2A) is apparently altered by ebselen, with its precise orientation appearing to depend on the conformation of ebselen (*i.e.*, **I** or **II**; Fig. 2B). Previous work has suggested that modification of Cys354 by an inhibitor (*e.g.*, carbapenems, penems) leads to conformational changes in the lid which stabilize the inhibitor-enzyme complex, thereby contributing to inhibitor potency.^10^,^28^ A comparison of our structures with reported Ldt_Mt2_ complex structures reveals variations in the conformation of the lid depending on the nature of the modification to Cys354 (Fig. 2C). It appears that the hydrophobic residues of the lid (*e.g.*, Tyr308, Met303, Tyr318, Thr320) can adjust to accommodate the group bonded to Cys354, apparently to optimise hydrophobic interactions. These results imply that the lid is conformationally dynamic, with the precise structure observed being related to the nature of modification of the nucleophilic Cys354. Thus, it seems likely that there is considerable scope for induced fit during catalysis and inhibition of Ldt_Mt2_, and by implication other Ldts.

The combined results demonstrate the potential of non-β-lactam compounds to inhibit Ldt_Mt2_*via* reaction with its nucleophilic cysteine, the probable mechanism for most, if not all of the compounds investigated. There is likely very considerable scope for application of this general mechanism for Ldt inhibition, perhaps building on efforts to target cysteine nucleophiles in proteases,^13^ cancer targets,^15^ and for chemical biology purposes.^20^ Of the cysteine-targeting reagents, ebselen was found to be the most potent against Ldt_Mt2_. Crystallographic analyses indicate that this potency relates to hydrophobic interactions involving the active-site lid of Ldt_Mt2_, and the conformation of this lid appears to depend on the nature of the modification to the nucleophile, Cys354. Whilst ebselen is almost certainly non-selective, there is clear potential for optimisation of the general approach. Overall, we hope the results presented here will help enable and inspire efforts to explore targeting the Ldts for TB treatment.

We are grateful to Dr Robert H. Bates for helpful discussions. This project was co-funded by the Tres Cantos Open Lab Foundation (Project TC 241). We thank the Wellcome Trust and the Medical Research Council (MRC) for funding. P. A. L. thanks the Medical Research Foundation (MRF) for support. T. R. M. thanks the Biotechnology and Biological Sciences Research Council (BBSRC) for support (grant number BB/M011224/1).

## Conflicts of interest

There are no conflicts to declare.

## Supplementary Material

Supplementary information
