# Supplementary material for: Targeting the Mycobacterium tuberculosis transpeptidase LdtMt2 with cysteine-reactive inhibitors including ebselen
Source: Chem Commun (Camb). 2019 Aug 5;55(69):10214–7. doi: 10.1039/c9cc04145a (PMC6984337; doi:10.1039/c9cc04145a)
Supplement: Supplementary file 1 [file CC-055-C9CC04145A-s001.pdf]

Electronic Supplementary Material (ESI) for ChemComm.
This journal is © The Royal Society of Chemistry 2019

Supporting Information for:

Targeting the *Mycobacterium tuberculosis* transpeptidase Ldt_{Mt2} with cysteine-reactive inhibitors including ebselen

Mariska de Munnik,[1 †] Christopher T. Lohans,[1] Pauline A. Lang,[1] Gareth W. Langley,[1 ‡] Tika R. Malla,[1] Anthony Tumber,[1] Christopher J. Schofield,[1*] and Jürgen Brem,[1*]

[1]Chemistry Research Laboratory, Department of Chemistry, University of Oxford, Oxford, OX1 3TA, United Kingdom.

[†] Current address: Tres Cantos Medicines Development Campus, GlaxoSmithKline, Severo Ochoa 2, Tres Cantos, Madrid, Spain

[‡] Current address: Charles River Laboratories, Chesterford Research Park, Saffron Walden, Essex, CB10 1XL, United Kingdom.

*Address correspondence to co-corresponding authors:

Prof. Christopher J. Schofield, Tel: +44 (0)1865 275625; Fax: +44 (0)1865 285002; Email: christopher.schofield@chem.ox.ac.uk

Dr. Jürgen Brem, Email: jurgen.brem@chem.ox.ac.uk

Supporting Information Contents

Experimental Details S3

Synthesis of **1** S6

Figure S1. Enzymatic activity of the L,D-transpeptidases S7

Figure S2. IC_{50} curves for inhibition of Ldt_{Mt2} by **2-11**. S8

Figure S3. MS analysis of covalent adduct formation of Ldt_{Mt2}. S9

Table S1. MS analysis of covalent adduct formation for Ldt_{Mt2} with **2-11** S10

Figure S4. Proposed reaction between Cys354 and thiol-reactive reagents. S11

Table S2. Processing and refinement statistics for Ldt_{Mt2} crystal structures S13

Figure S5. Electron density maps for the Ldt_{Mt2}:ebselen complex S14

References S15

Experimental Details

General

Faropenem was from Selleckchem, fluorescein was from Fisher, and all other chemicals were from Sigma Aldrich. Chemicals were used without further purification, unless stated otherwise. Column purifications were carried out using a Biotage Isolera One system unless otherwise specified. TLC was used to monitor conversions and was performed using TLC silica gel 60 F254 sheets from Merck. NMR characterisation of synthesised compounds was performed using a 2-channel Bruker AVIIIHD 400 MHz Nanobay instrument. High-resolution mass spectrometry (HRMS) characterisation was performed using a Thermo Exactive mass spectrometer equipped with a UPLC system.

Protein Production

Ldt_{Mt2} Δ1-55 was produced and purified as described previously.[1] In summary, a culture of *Escherichia coli* BL21(DE3) cells transformed with the pNIC28-Bsa4-Ldt_{Mt2} Δ1-55 vector was grown in 2TY media (with 50 μg/mL kanamycin). Protein production was induced when the culture OD_{600} reached 0.6 through the addition of 0.5 mM isopropyl β-D-thiogalactopyranoside (IPTG); the culture was then incubated at 18 °C with shaking (180 rpm) overnight. Following cell lysis via sonication, Ldt_{Mt2} Δ1-55 was purified by chromatography using a 5 mL HisTrap column (GE Life Sciences) and a 300 mL Superdex 200 column (GE Life Sciences). Purified Ldt_{Mt2} Δ1-55 (in 50 mM Tris, pH 8.0, 100 mM NaCl) was frozen on liquid nitrogen and stored at -80 °C. For crystallographic studies, the His-tag was cleaved with tobacco etch virus (TEV) protease. The purity and identity of Ldt_{Mt2} Δ1-55 was confirmed by SDS-PAGE (>95 % purity) and mass spectrometry (calculated 40444 Da, observed deconvoluted mass 40441 Da).

Fluorogenic Assay

Inhibition assays were performed in flat-bottomed 384-well μ-clear plates (Greiner Bio-One) with a final volume of 25 μL, and using 50 mM HEPES, pH 7.5 containing 0.01% (v/v) Triton X-100 as the assay buffer. Ldt_{Mt2} (100 nM) was treated with a range of inhibitor concentrations, and the mixture was incubated for 0, 10 or 60 minutes. Following this pre-incubation, fluorogenic probe **1** (25 μM)[2] was added, and the mixture was incubated for a further 30 minutes. The fluorescence signal was then measured using a BMG Labtech PHERAstar FS with λ_{ex} = 480 nm and λ_{em} = 520 nm using the bottom optic. All reactions were carried out in quadruplicate, and controls without inhibitor and without Ldt_{Mt2} were included. Inhibition results were normalised using the no inhibitor and no enzyme controls. Dose-response analysis was performed using Prism (GraphPad) with the log(inhibitor) vs normalized response -- variable slope model. Data points were plotted as mean average with the standard deviation as the error bars.

Mass Spectrometry

Mass spectrometric analyses were performed using an Agilent 6550 system equipped with a RapidFire sampler. Samples (100 μL) were prepared in 50 mM Tris, pH 7.5, consisting of 3 μM Ldt_{Mt2} and either 6 μM or 30 μM of the relevant inhibitor, as specified elsewhere.

X-ray Crystallography

Purified Ldt_{Mt2} Δ1-55 (approx. 1 mM; His-tag cleaved) in 50 mM Tris, pH 8.0, 100 mM NaCl was used for crystallography. Crystallisation trials were set up using the sitting drop vapour diffusion method with the JCSG+ broad screen (Molecular Dimensions). A structure of Ldt_{Mt2} Δ1-55 (PDB 6RLG) was solved using a crystal

obtained from a well solution consisting of 0.2 M ammonium nitrate and 20 % w/v PEG 3350. A structure of Ldt_{Mt2} Δ1-55 in complex with ebselen (PDB 6RRM) was solved by soaking the crystals obtained above with 15 mM ebselen. Data were collected at 100 K at the Diamond Light Source synchrotron IO4-1 beamline. Data were indexed and integrated with XDS, and scaled using SHELX.[3] Phaser was used to solve the structure by molecular replacement,[4] using PDB entry 5DU7 as a search model.[5] Fitting and refinement were carried out using COOT[6] and PHENIX[7] until R_{work} and R_{free} no longer converged. The final statistics for data collection and refinement are listed in Table S2.

Synthesis of 2-(6-(((2,4-dinitrophenyl)sulfonyl)oxy)-3-oxo-3H-xanthen-9-yl)benzoic acid (1)[2]

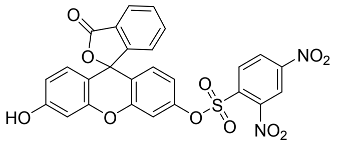

Reactions were shielded from light during all stages of the synthesis. Fluorescein (665 mg, 2.00 mmol, 1.0 eq) was suspended in dichloromethane (DCM; 20 mL), and the temperature was reduced to 0 °C. Triethylamine (0.340 mL, 2.40 mmol, 1.2 eq) was then added and the reaction mixture was stirred for 5 minutes, then 2,4-dinitrobenzenesulfonyl chloride (533 mg, 2.40 mmol, 1.2 eq) was added. The reaction mixture was allowed to warm to room temperature and was stirred for 5.5 hours. DCM (60 mL) was added and the mixture was washed with HCl (1.0 M, 3 x 60 mL) and brine (60 mL). The organic layer was dried over magnesium sulphate, filtered and solvents were evaporated under reduced pressure. The crude product was purified using column chromatography (cHex/EtOAc 100:0 to 60:40, 2x). **1** was obtained as a yellow solid (0.558 g, 50%). ^{1}H NMR (400 MHz, DMSO-d_6; mixture of quinone and spirolactone forms)[8] δ 10.35 – 10.16 (m, 1H), 9.18 – 8.88 (m, 1H), 8.65 – 8.48 (m, 1H), 8.37 (d, J = 8.6 Hz, 1H), 8.12 – 7.97 (m, 1H), 7.89 – 7.71 (m, 2H), 7.45 – 7.31 (m, 2H), 7.07 – 6.86 (m, 3H), 6.76 – 6.56 (m, 2H). ^{13}C NMR (400 MHz, DMSO; mixture of quinone and spirolactone forms)[8] δ 168.61, 168.36, 160.00, 155.84, 152.35, 151.79, 151.56, 151.43, 150.88, 149.59, 149.42, 148.28, 136.10, 133.79, 130.90, 130.65, 130.56, 130.45, 129.93, 129.31, 127.89, 125.77, 125.40, 125.10, 124.21, 122.14, 121.38, 121.20, 119.39, 118.80, 118.65, 117.90, 116.72, 113.59, 111.08, 110.92, 109.04, 102.47, 81.59, 80.07. HRMS (ESI$^+$) $C_{26}H_{15}O_{11}N_2{}^{32}S$ ($[M+H]^+$) observed 563.0392, calculated 563.0318.

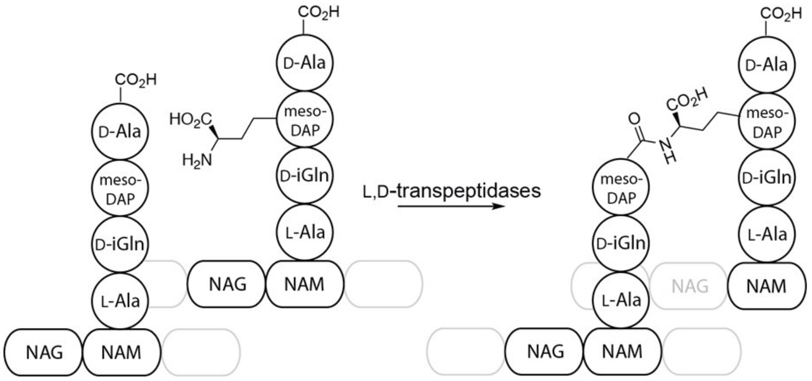

Figure S1. Enzymatic activity of the L,D-transpeptidases.[9] Scheme showing 3→3 peptide cross-link formation in the mycobacterial cell wall, as catalysed by the L,D-transpeptidases. *meso*-Dap: *meso*-diaminopimelate; iGlu: isoglutamine; NAG: *N*-acetylglucosamine; NAM: *N*-acetylmuramic acid.

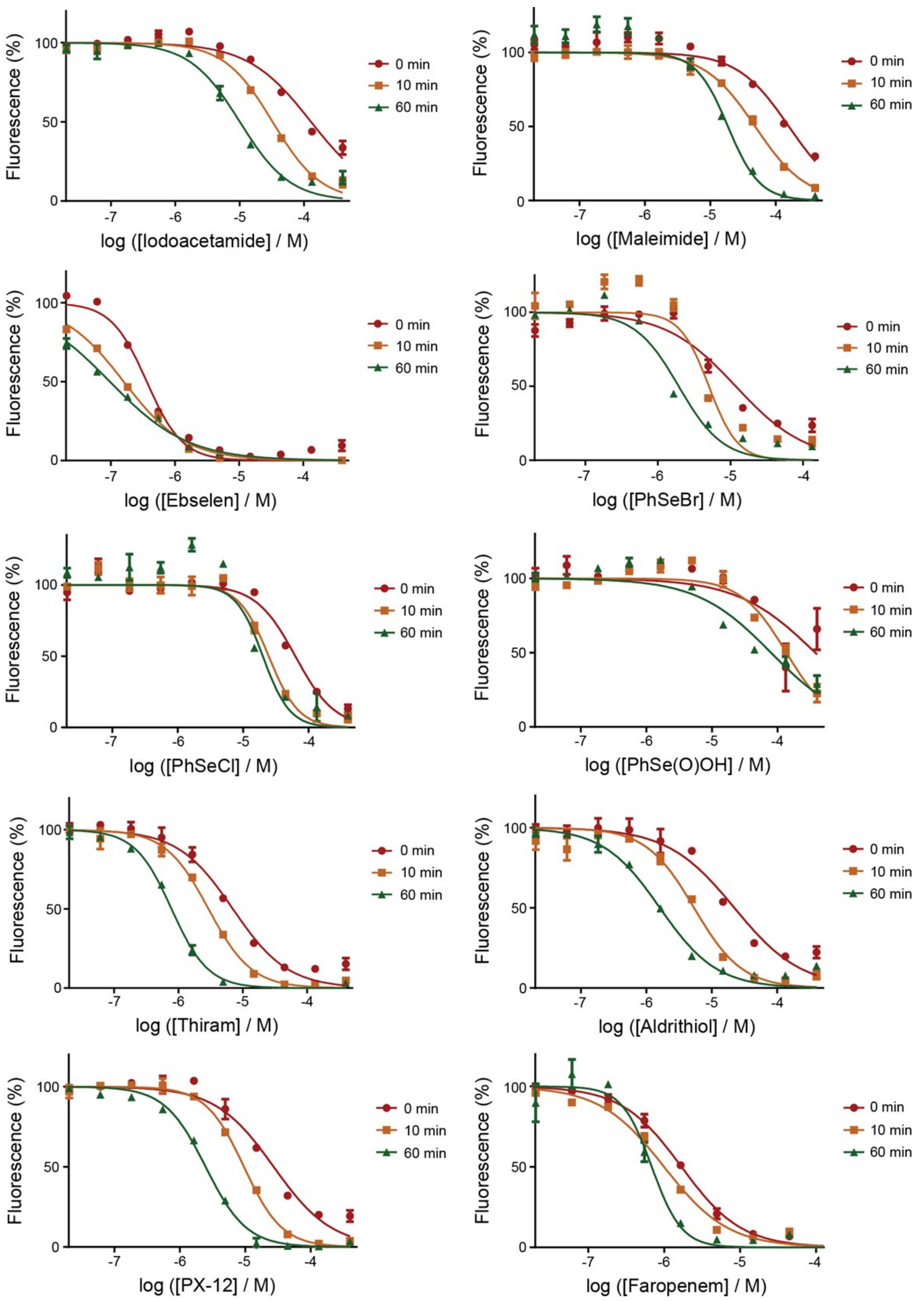

Figure S2. IC_{50} curves for inhibition of Ldt_{Mt2} by **2-11** after 0, 10 and 60 minutes of pre-incubation. The assay conditions are described in the Experimental Details section.

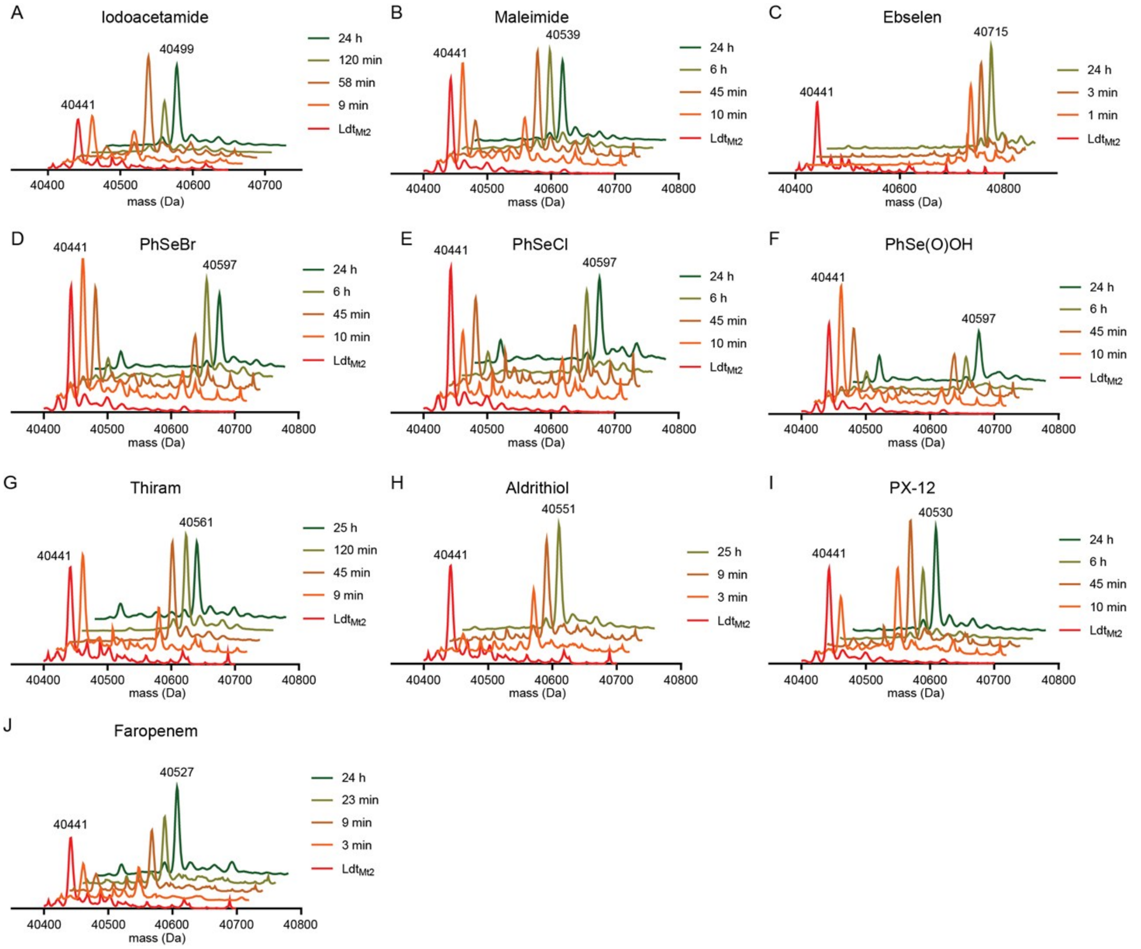

Figure S3. MS analysis of the apparent covalent adducts formed with Ldt$_{Mt2}$ and the cysteine-reactive inhibitors. Ldt$_{Mt2}$ (3 µM) was treated with 6 µM of (A) iodoacetamide, (B) maleimide, (C) ebselen, (D) PhSeBr, (E) PhSeCl, (F) PhSe(O)OH, (G) thiram, (H) aldrithiol, (I) PX-12, and (J) faropenem. Covalent adducts with Ldt$_{Mt2}$ were observed for all of the tested compounds, although their rates of formation differed. The conditions used for mass spectrometry are described in the Experimental Details section. The mass shifts associated with the adducts are summarised in Table S1, while unmodified Ldt$_{Mt2}$ was observed with a deconvoluted mass of 40441 Da.

Table S1. Mass spectrometric analysis of covalent adduct formation for Ldt_{Mt2} with compounds **2-11**.

Compound		Molecular weights of adducts		
		Observed deconvoluted mass (Da)	Observed mass shift (Da)	Calculated mass shift (Da)
	Ldt_{Mt2}	40441[a]	-	-
**2**	Iodoacetamide	40499	58	58
**3**	Maleimide	40539	98	98
**4**	Ebselen	40715	274	274
**5**	PhSeBr	40597	156	156
**6**	PhSeCl	40597	156	156
**7**	PhSe(O)OH	40597	156	156
**8**	Thiram	40561	120	120
**9**	Aldrithiol	40551	110	110
**10**	PX-12	40530	89	89
**11**	Faropenem[b]	40527	86	86

[a]Calculated mass: 40444 Da.

[b]The observed mass shift for the adduct formed with faropenem is consistent with previous reports.[1,5]

A

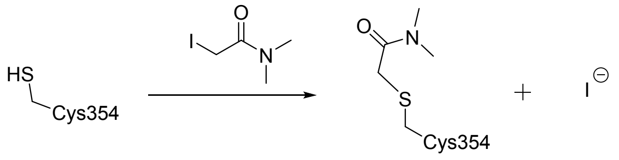

B

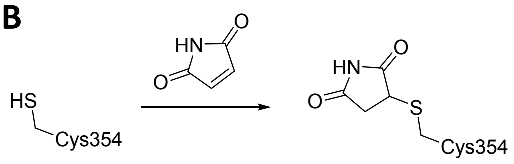

C

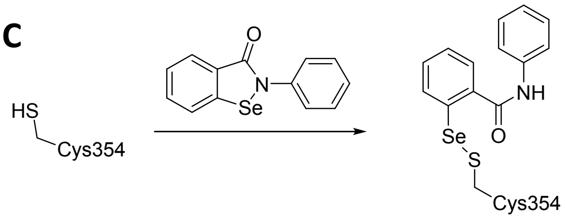

D

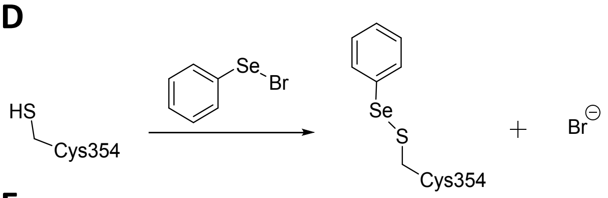

E

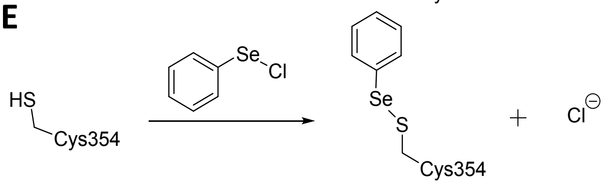

F

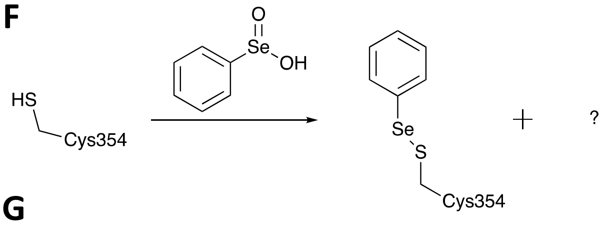

G

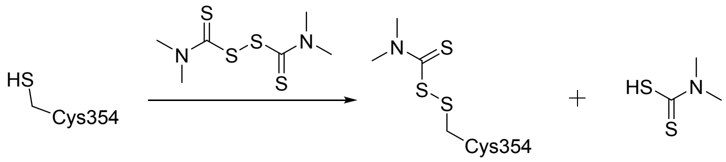

H

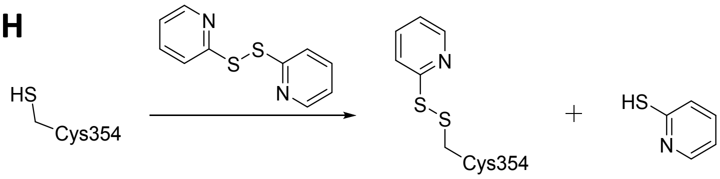

I

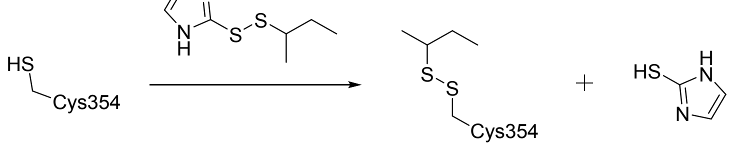

Figure S4. Proposed reactions between the nucleophilic cysteine of Ldt_{Mt2} and the thiol reactive reagents, with likely product structures. The structures shown for the products derived from (A) iodoacetamide, (B) maleimide, (C) ebselen, (D) PhSeBr, (E) PhSeCl, (F) PhSe(O)OH, (G) thiram, (H) aldrithiol, and (I) PX-12 are supported by adducts observed by mass spectrometry (Figure S3). Note that the products formed by the reaction of Ldt_{Mt2} with PhSe(O)OH are presently unknown.

Table S2. Processing and refinement statistics for Ldt_{Mt2} crystal structures.

Data Set	Ldt_{Mt2} Apo	Ldt_{Mt2}:Ebselen complex
**PDB**	6RLG	6RRM
**Resolution (outer shell) (Å)**	32.93 - 1.51 (1.564 - 1.51)	48.75 - 1.64 (1.699 - 1.64)
**Unit cell dimensions**	60.95 93.92 75.57<br>90 93.03 90	61.22 93.57 75.49<br>90 93.01 90
**Space group**	P 1 21 1	P 1 21 1
**Protein molecules per ASU†**	2	2
**Completeness (outer shell) (%)**	99.80 (99.87)	99.84 (99.95)
**No. of unique reflections (outer shell)**	132752 (13249)	103733 (10319)
**Multiplicity (outer shell)**	6.54 (6.71)	6.65 (6.81)
**CC-half**	0.9987 (0.5603)	0.9985 (0.5364)
**R_{merge}**	0.07	0.0696
**I/σ mean (outer shell)**	12.03 (1.08)	12.09 (1.05)
**Wilson B**	21.26	27.25
**Refinement**		
**B factors:**		
**Overall**	28.46	37.54
**Protein**	25.97	36.87
**Ligand**	52.11	54.40
**Water**	41.76	42.38
**RMSD from ideal bond length (Å)‡**	0.009	0.006
**RMSD from ideal angles (degrees)**	0.99	0.81
**R_{work} (%)**	18.02	20.58
**R_{free} (%)**	20.38	22.85

†ASU = asymmetric unit. ‡RMSD = root mean square deviation.

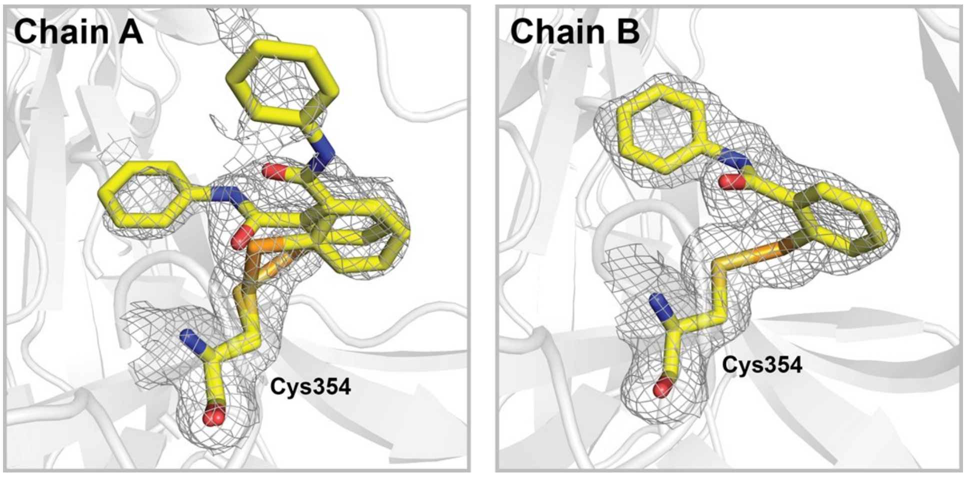

Figure S5. Electron density maps for the complex derived from Ldt_{Mt2} and ebselen. The electron density corresponding to the covalent adduct of ebselen with Cys354 is represented as a 2mFo-DFc map (contoured at 1σ) for chains A and B of PDB entry 6RRM.

References

1 C. T. Lohans, H. T. H. Chan, T. R. Malla, K. Kumar, J. J. A. G. Kamps, D. J. B. McArdle, E. van Groesen, M. de Munnik, C. L. Tooke, J. Spencer, R. S. Paton, J. Brem, and C. J. Schofield, *Angew. Chem. Int. Ed. Engl.* 2019, **131**, 2012-16.

2 H. Maeda, H. Matsuno, M. Ushida, K. Katayama, K. Saeki, and N. Itoh, *Angew Chem Int Ed Engl* 2005, **44**, 2922-25.

3 G. M. Sheldrick, *Acta Cryst.* 2008, **A64**, 112-22.

4 A. J. McCoy, R. W. Grosse-Kunstleve, P. D. Adams, M. D. Winn, L. C. Storoni, and R. J. Read, *J. Appl. Crystallogr.* 2007, **40**, 658-74.

5 P. Kumar, A. Kaushik, E. P. Lloyd, S. G. Li, R. Mattoo, N. C. Ammerman, D. T. Bell, A. L. Perryman, T. A. Zandi, S. Ekins, S. L. Ginell, C. A. Townsend, J. S. Freundlich, and G. Lamichhane, *Nat Chem Biol* 2017, **13**, 54-61.

6 P. Emsley, B. Lohkamp, W. G. Scott, and K. Cowtan, *Acta Crystallogr. D Biol. Crystallogr.* 2010, **66**, 486-501.

7 P. D. Adams, P. V. Afonine, G. Bunkóczi, V. B. Chen, I. W. Davis, N. Echols, J. J. Headd, L. W. Hung, G. J. Kapral, R. W. Grosse-Kunstleve, A. J. McCoy, N. W. Moriarty, R. Oeffner, R. J. Read, D. C. Richardson, J. S. Richardson, T. C. Terwilliger, and P. H. Zwart, *Acta Crystallogr. D Biol. Crystallogr.* 2010, **66**, 213-21.

8 W. R. Orndorff and A. J. Hemmer, *J. Am. Chem. Soc.* 1927, **49**, 1272-80.

9 M. Lavollay, M. Arthur, M. Fourgeaud, L. Dubost, A. Marie, N. Veziris, D. Blanot, L. Gutmann, and J. L. Mainardi, *J Bacteriol* 2008, **190**, 4360-66.
